# Knowledge and practices regarding infection control precautions against blood-borne diseases among recovered HCV patients in Egypt

**DOI:** 10.1038/s41598-025-23618-3

**Published:** 2025-10-31

**Authors:** Mohamed Fakhry Hussein, Wesal Youssef Hassan, Mohamed Hossam Mohamed, Marwa Mostafa Mohamed, Hossam Mohamed Hassan Soliman

**Affiliations:** 1 Department of Occupational Health and Industrial Medicine, High Institute of Public Health, Alexandria University, Alexandria, Egypt; 2 Clinical Research Department, Health Insurance Organization (North West Area), Alexandria, Egypt; 3 Biotechnology Department, Faculty of Science, Helwan National University, Cairo, Egypt; 4 Clinical Governance Department, Health Insurance Organization (North West Area), Alexandria, Egypt; 5 Faculty of Applied Health Sciences, Borg El-Arab Technological University, Alexandria, Egypt

**Keywords:** Hepatitis c virus, Blood-borne diseases, Knowledge, Practices, Egypt, Infection control, Gastroenterology, Health care, Risk factors

## Abstract

Preventing Hepatitis C Virus (HCV) reinfection is vital for the long-term health of recovered patients. This cross-sectional study evaluated the knowledge and practices related to blood-borne disease infection control among 376 HCV-recovered individuals in Egypt. Participants had a median age of 63 years, 81.9% were males, 56.1% were manual workers, 6.9% were healthcare professionals, 67.6% had basic literacy, and only 47.9% had received the hepatitis B vaccine. Non-healthcare participants had significantly poorer knowledge and practices compared to healthcare professionals (*p* < 0.001). Both groups showed suboptimal levels, with 19.2% of healthcare professionals and 0.3% of non-healthcare participants demonstrating good knowledge, while good practices were observed in 23.0% and 14.3% of each group, respectively. A significant positive correlation existed between knowledge and practice (*r* = 0.363, *p* < 0.001). Higher education (*p* < 0.01) and the HBV vaccination status (*p* = 0.004) were associated with better knowledge. Higher education correlated with better practices (*p* < 0.05). Individuals in manual labor had lower knowledge and practice scores (*p* = 0.012 and *p* = 0.001, respectively). These findings underscore significant knowledge and practice deficits, emphasizing the need for targeted interventions to improve infection control and sustain the success of Egypt national HCV treatment program.

**Supplementary Information:**

The online version contains supplementary material available at 10.1038/s41598-025-23618-3.

## Introduction

Hepatitis C is a liver infection caused by the hepatitis C virus (HCV). The virus can lead to both acute and chronic hepatitis, ranging from mild, self-limited symptoms to a lifelong condition that can cause liver cirrhosis or cancer. Globally, an estimated 58 million people are infected with HCV, with about 1.5 million new cases reported annually. Most hepatitis C infections are transmitted through exposure to contaminated blood, such as from unsafe injection practices, risky medical procedures, unscreened blood transfusions, sharing needles, and certain sexual behaviors^1,2^.

While direct-acting antiviral medications (DAAs) can cure over 95% of people with hepatitis C, access to diagnosis and treatment remains limited in many areas. In 2015, Egypt had one of the highest rates of HCV infection worldwide, with 7% of adults affected. Between 2014 and 2020, the country launched a massive nationwide campaign concerning HCV infection, which is considered the largest HCV management project globally. Through this campaign, Egypt screened more than 50 million people for HCV and treated approximately 4 million patients with the new DAAs. These efforts successfully reduced HCV prevalence in Egypt to just 0.38% by 2023, making it the first country to attain the World Health Organization (WHO) “gold tier” status on the way to hepatitis C elimination^2–4^.

The WHO 2030 elimination strategy for viral hepatitis aims to drastically reduce new infections and deaths from hepatitis B and C. This ambitious global health goal targets a 90% reduction in new chronic infections and a 65% reduction in mortality. Key to achieving this is increasing access to vaccination, expanding testing and diagnosis, and ensuring all people with chronic hepatitis B and C have access to life-saving treatment. The strategy focuses on a public health approach, emphasizing prevention and integrated care to make elimination a reality^5^.

The Egyptian Ministry of Health and Population (MOHP) offered the DAAs to every person with a confirmed diagnosis of HCV infection at a very low price^4^. This reduction in the incidence and prevalence of HCV promotes health and reduces the money spent on treating and supporting cases in the long run. A study in 2023 carried out by Ezzat et al. applied a cost-effectiveness model to examine the impacts of the Egyptian HCV national campaign on the future. They concluded that by 2030, Egypt could save about $35 million in direct costs and $4705 million in indirect costs^6^.

Recurrence of HCV infection after treatment could be due to non-compliance with treatment, infection with different strains of HCV, as some strains are more difficult to treat than others, a weakened immune system, especially in those with HIV, cancer, after liver transplantation, or reinfection due to being exposed to the virus again. The reinfection can happen through contact with blood or other bodily fluids from an infected person^7^.

Infection prevention and control (IPC) are the practices and procedures used to prevent the spread of infection. They are used in healthcare settings and other settings, such as schools, childcare facilities, and home healthcare. The Centers for Disease Control and Prevention (CDC) defines infection control precautions as “the collective measures used to prevent the spread of infection.” These measures include hand hygiene, as washing hands with soap and water is the single most important way to prevent the spread of infection. Besides, environmental cleaning and disinfection procedures are carried out to kill pathogenic microorganisms. Safe injection practices are also important through using sterile needles and syringes and avoiding needlestick injuries. Using personal protective equipment (PPE), such as wearing gloves, gowns, masks, and eye protection, can help protect against exposure to blood and other body fluids. Blood and other body fluids must be tested before handling these subjects^8,9^.

Infection prevention and control for HCV patients or HCV-recovered people should include practicing good hand hygiene, especially after having contact with blood or other body fluids; using gloves when there is a risk of exposure to blood or other body fluids; and avoiding sharing personal care items such as razors, toothbrushes, nail clippers, and other items that could come into contact with blood^7–9^. Following the successful national campaign to treat hepatitis C, preventing reinfection is crucial. By doing so, the efforts and resources invested in their recovery can be preserved, ensuring that recovered individuals remain healthy, productive, and free from the stigma associated with HCV.

## Methods

### Aim of the study

This study aimed to assess knowledge and practices of people who have recovered from HCV regarding infection control precautions against blood-borne diseases, including the proper handling of sharps and blood to avoid the recurrence of the infection. Besides, it investigated their socio-demographic characteristics.

### Study design and setting

A cross-sectional study using interview questionnaires was conducted in government healthcare units in Alexandria, Egypt, from January 15th to August 15th, 2024.

### Target population

Participants were eligible to participate if they were over 18 years old, had recovered from HCV, and were attending healthcare units for follow-up on the recovery condition. Pregnant women and individuals under 18 years old were excluded from the study.

### Sample size and method of selection

Assuming 54% of participants had good knowledge of infection control precautions for blood-borne diseases^10^, with a 5% margin of error, a 95% confidence interval, and 80% study power with a population size (attending purposed healthcare facilities) of 30,710, a minimum sample size of 376 was calculated using Epi-Info software. This sample size was collected using a non-random sampling method, combining convenience and snowball sampling techniques.

### Data collection tools

A structured interview questionnaire was adapted from previously validated questionnaires and after an extensive review of the literature^11–16^. The questionnaire reliability was assessed using Cronbach’s alpha test, which was 0.774, indicating high reliability with consistent responses^17^. To ensure face and content validity, an expert committee (consisting of three professors in public health, internal medicine, and infection prevention and control) reviewed the tool. They evaluated if it measured the intended construct and covered all relevant aspects. The committee suggested minor modifications for clarity and scientific accuracy. A pilot study was then conducted to confirm the face validity.

The questionnaire had three sections (Supplementary File I). The **first section** asked about socio-demographic information, including age, sex, nationality, residence, marital status, education, and occupation. It also included questions about sources of information on infection control precautions against blood-borne diseases, the duration of HCV recovery, whether the participant had received the hepatitis B virus (HBV) vaccine, and whether they had experienced any injuries from sharp instruments within the past year.

The **second section** assessed the knowledge about infection control precautions against blood-borne diseases by asking questions about the definition of blood-borne diseases, sources of infection, preventive measures, and infection control standard precautions. The knowledge was assessed using 15 questions, which include yes, no, or I do not know options. A correct answer received a ‘1’ score, and an incorrect answer received a ‘0’ score. “I don’t know” was considered a wrong answer and given a zero score. The total score was converted to a percentage that ranged from 0 to 100%. The knowledge level scoring system was categorized into three levels: poor level (0 - < 50%), fair level (50- <70%), and good level (70–100%)^18–21^.

The **third section** evaluated the practices of the participants concerning dealing with sharps or blood and infection control precautions. Twelve questions were asked to assess the frequency of certain practices related to infection control precautions for blood-borne diseases and actions on exposure to needle stick injury. To assess the practices, a Likert 5-point scale was used. For good or sufficient practice, the score was 5 for the “always” answer, 4 for “usually,” 3 for “sometimes,” 2 for “rarely,” and 1 for the “never” response. However, the reverse score was given for bad practices. The total score was converted to a percentage and was categorized into three levels: poor practice (0 - < 50%), fair practice (50- <70%), and good practice (70–100%)^18,20^.

### Pilot study

A pilot study was conducted before the main study to evaluate the survey practicality, clarity of questions, and the time required to complete it (approximately 15–20 min). Twenty participants were recruited for the pilot study, and some minor revisions were made to clarify vague wording. However, these pilot participants were not included in the final analysis.

### Ethical considerations

The research was approved by the Research Ethics Committee of the Ministry of Health and Population (OHRP: FWA00016183, IORG0005704/IRB0000687, Date: 6/12/2023). It followed the International Guidelines for Research Ethics outlined in the 1964 Declaration of Helsinki and its subsequent amendments. Participants were informed about the research purpose at the beginning of the questionnaire and provided written consent before participating. Each question was asked in the same manner for all participants, and any unclear questions were clarified. Participants had the right to withdraw from the survey at any time. Anonymity and confidentiality were ensured throughout the research.

### Statistical analysis

The collected data were entered into an Excel sheet to be analysed using SPSS software version 26. The Shapiro-Wilk test was used to assess the normality of the data. For continuous variables, normally distributed data were presented as mean ± standard deviation (SD), while not-normally distributed data were presented as median and interquartile range (IQR), besides minimum/maximum values. Categorical variables were described using frequencies and percentages. We used the chi-square test (χ^2^) to compare categorical variables (levels of knowledge and practices) between different groups. The Mann-Whitney (U) test was employed to compare contanious variables (knowledge and practice scores) as the data were not normally distributed between two independent groups.

The Spearman correlation coefficient (r) was used to measure the strength and direction of the relationship between two variables when the data is not normally distributed. Cronbach’s alpha test was used to assess the reliability of the study tool. Finally, the generalized linear model regression analyses were used to identify factors influencing knowledge and practice of infection control precautions related to blood-borne diseases, as the data were not normally distributed and did not meet the assumptions of ordinary least squares regression. This approach accommodates the skewness and heteroscedasticity observed in the data and enables simultaneous adjustment for multiple predictors, allowing more transparent estimate of the associations between the independent variables and participants’ knowledge and practice. The Omnibus test was used to determine if the overall model significantly improves the prediction of the dependent variables (knowledge and practice) compared to a model with no predictors. A *p*-value (*p*) less than 0.05 was considered statistically significant for all inferential analyses.

## Results

### Socio-demographic characteristics, HBV vaccination, and duration of HCV recovery of the study population

**Fig. 1:**
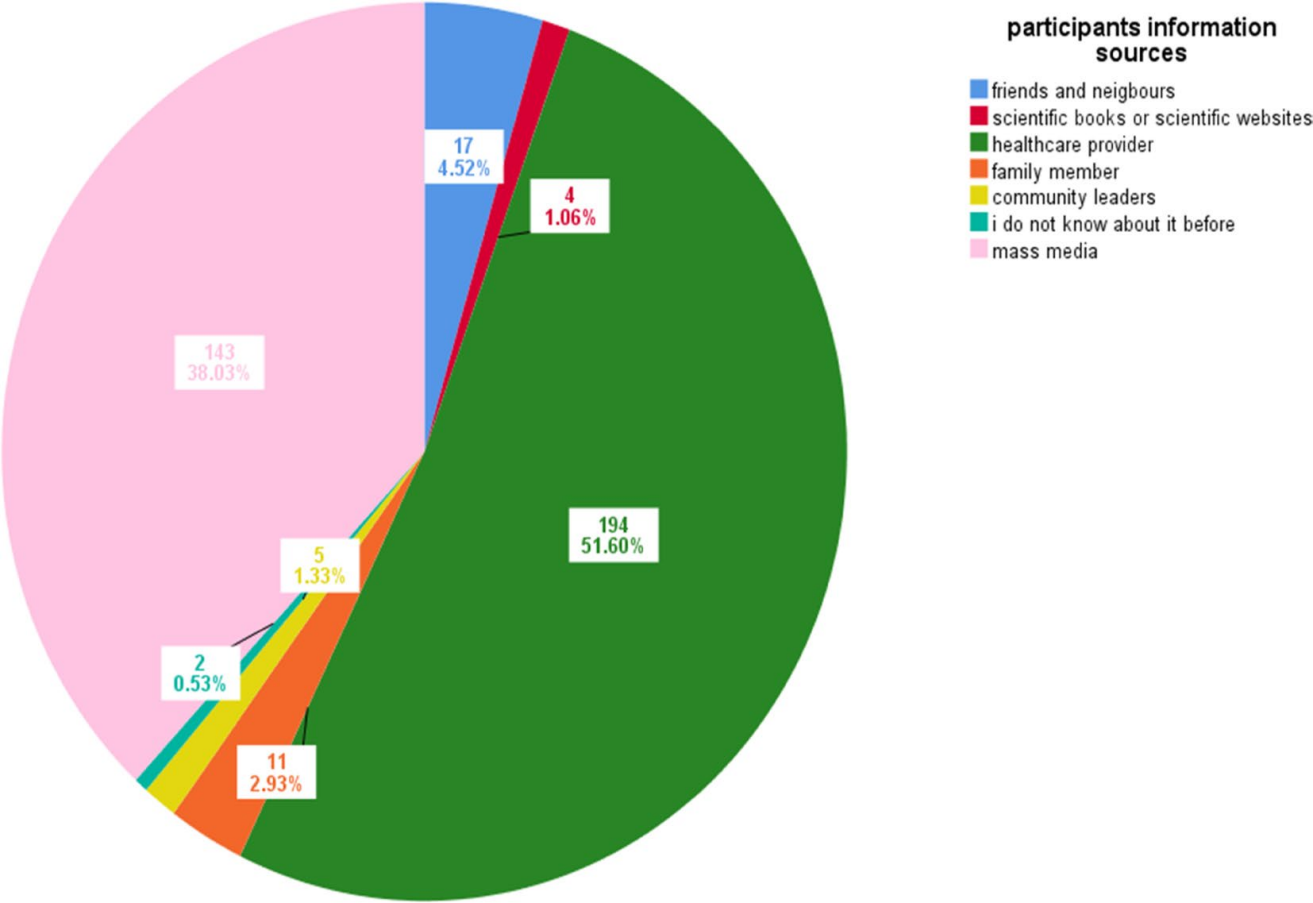
Pie chart for source of information about infection control precautions towards blood-borne diseases, 2024 (*n* = 376).

Table 1 presents the socio-demographic characteristics of the 376 study participants. The median age of the study sample was 63 years. The majority of participants were male (81.9%), resided in Alexandria (97.0%), and lived in urban areas (90.2%). Regarding educational level, about two-thirds of the respondents can only read and write (67.6%), and 14.1% had secondary education. Most of them were married (82.8%). In terms of employment status, 24.7% were unemployed, 12.3% were employed in professional occupations such as engineers and chemists, 6.9% were healthcare professionals, and 56.1% were employed in manual labor occupations such as fishermen, farmers, plumbers, and factory workers. Nearly half of the participants (47.9%) had received the hepatitis B vaccine, and 40.7% had recovered from HCV within 2–4 years. The majority of the participants had not been exposed to injury by sharp instruments within the last year (91.2%). However, 8.8% of participants reported experiencing one or more needle-stick injuries within the past year. Healthcare providers (51.6%) were the primary source of information on infection control precautions against blood-borne diseases, followed by media (38.0%) (Fig. 1).

**Table 1:** Socio-demographic characteristics, HBV vaccination, and duration of HCV recovery of the study population, 2024 (*n* = 376).

Socio-demographic characteristics	No.	%
Age (years)		
• Median (IQR)		63 (57–68)
• Min-Max		27–85
Gender		
• Male	308	81.9
• Female	68	18.1
Living governorate		
• Alexandria	365	97.0
• Behera	10	2.7
• Marsa Matrouh	1	0.3
Nature of residence		
• Urban	339	90.2
• Rural	37	9.8
Education level		
• Read and write	254	67.5
• Primary school	39	10.4
• Secondary school	53	14.1
• Bachelor’s degree	26	6.9
• Postgraduate studies	4	1.1
Occupation		
• Non-healthcare Professional job	46	12.3
• Healthcare professionals	26	6.9
• Manual job	211	56.1
• Not working	93	24.7
Marital status		
• Married	309	82.2
• Not married	67	17.8
Took the HBV vaccine		
• Yes	180	47.9
• No	99	26.3
• I don’t know	97	25.8
Duration of HCV recovery		
• Less than 2 years	95	25.3
• From 2–4 years	153	40.7
• 5 years and more	128	34.0
Number of injuries by sharp instruments within the last year		
• Zero	343	91.2
• 1–2 times	14	3.7
• 3–10 times	13	3.5
• More than 10 times	6	1.6

Min: Minimum, Max: Maximum, IQR: Interquartile Range.

### Knowledge of the participants regarding blood-borne diseases

**Fig. 2:**
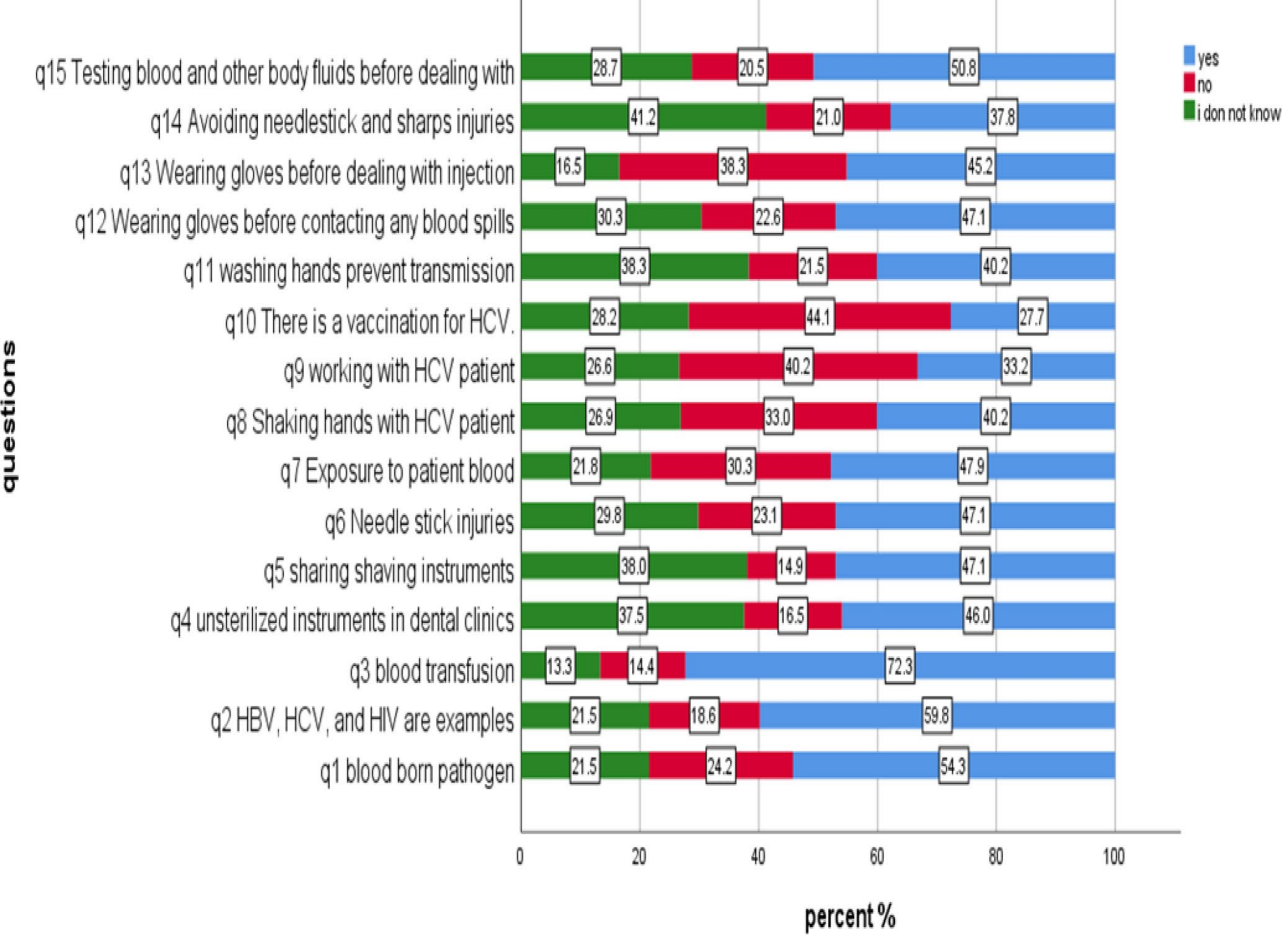
Likert plot illustrates the percentage of the response of the participants to the knowledge questions, 2024 (*n* = 376).

Figure 2 illustrates the participants’ responses to each question within the knowledge assessment section of the questionnaire. Correct answer rates ranged from approximately 40% to 60% across the questions. The highest percentage of correct answers was observed for question 3 (Blood transfusion could transmit blood-borne infections), with 72.3% of participants answering correctly. The lowest percentages of correct answers were observed for questions q8 (33.0%), q9 (40.2%), q10 (44.1%), q11 (40.2%), and q14 (37.8%). (For more details about the questions, refer to Supplementary File II).

### The participants’ practices regarding infection control precausions of blood-borne diseases

**Fig. 3:**
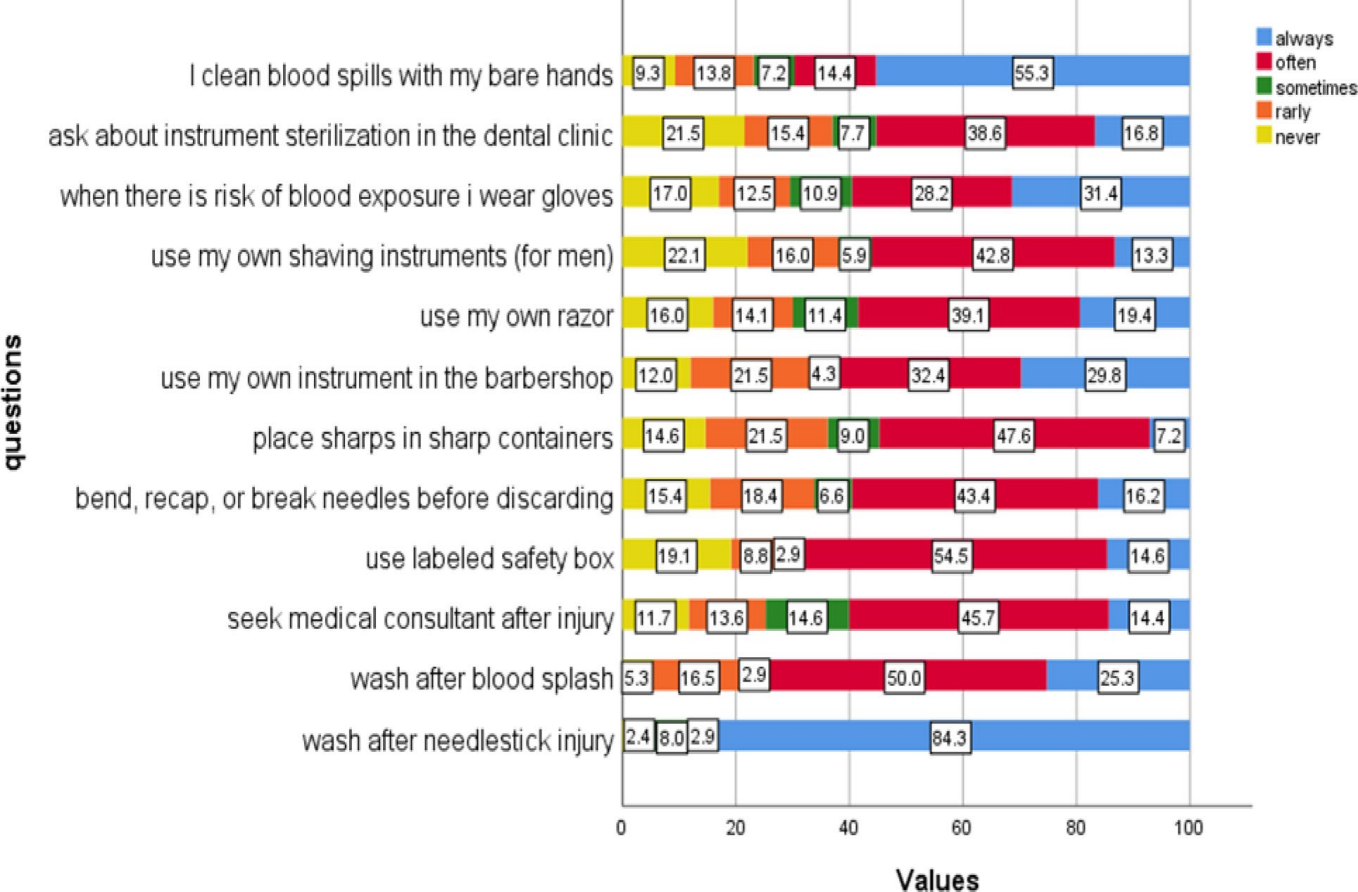
Likert plot shows the percentage of the response of the participants to the practice questions, 2024 (*n* = 376).

Figure 3 shows the responses of the participants to each question in the practice part of the questionnaire. The highest adherence to recommended practices was observed for washing the injured area with soap and water after a needle stick injury (84.3% of participants reported always doing so). In contrast, the lowest adherence to safe practices was observed for proper sharps disposal and safe blood spill cleanup. Only 7.2% of participants consistently placed sharps in designated containers immediately after use, and a concerning 9.3% reported never cleaning blood spills with bare hands. (For more details about the questions, refer to Supplementary File II).

### The diffecrence between healthcare professionals and non-healthcare participants regarding overall knowledge and practice

Table 2 summarizes the difference of overall knowledge and practice scores between healthcare professionals and non-healthcare participants regarding infection control precautions for blood-borne diseases. Non-healthcare participants showed significantly poorer knowledge and practice of infection control compared to healthcare professionals. Specifically, 78.5% of non-healthcare participants had poor knowledge, in contrast to just 11.5% of healthcare professionals (χ^2^ = 290.95, *p* < 0.001). Similarly, 66.0% of non-healthcare participants exhibited poor practices, while 30.8% of healthcare professionals did (χ^2^ = 25.74, *p* < 0.001). The median score percentages further highlight this gap, with healthcare professionals scoring much higher in both knowledge (68.1% vs. 43.7%; U = 18.69, *p* < 0.001) and practice (62.5% vs. 33.3%; U = 5.64, *p* = 0.018). While healthcare professionals performed better, it is noteworthy that their knowledge and practice levels were still suboptimal, as only a small percentage showed good knowledge (19.2%) and good practices (23.0%).

**Table 2:** Comparison of knowledge and practice scores regarding infection control precautions between healthcare professionals and other participants (*n* = 376), 2024.

Category	Non-healthcare participants (*n* = 350)	Healthcare professionals (*n* = 26)	Statistical test (*p*)
**Knowledge**			
Poor knowledge (< 50%)	275 (78.5%)	3 (11.5%)	χ^2^ = 290.95 (< 0.001*)
Fair knowledge (50–<70%)	74 (21.2%)	18 (69.3%)	
Good knowledge (≥ 70%)	1 (0.3%)	5 (19.2%)	
Knowledge score percentage (Min–Max)	14.5–82.0	6.25–93.7	
Knowledge score percentage (Mean ± SD)	45.7 ± 22.0	61.3 ± 22.3	
Knowledge score percentage (Median [IQR])	43.7 (31.2–62.5)	68.1 (62.5–75.0)	U = 18.69 (< 0.001*)
**Practice**			
Poor practice (< 50%)	231 (66.0%)	8 (30.8%)	χ^2^ = 25.74 (< 0.001*)
Fair practice (50–<70%)	69 (19.7%)	12 (46.2%)	
Good practice (≥ 70%)	50 (14.3%)	6 (23.0%)	
Practice score percentage (Min–Max)	1.0–83.3	11.4–83.3	
Practice score percentage (Mean ± SD)	36.6 ± 21.0	54.1 ± 24.0	
Practice score percentage (Median [IQR])	33.3 (16.6–52.0)	62.5 (33.3–68.7)	U = 5.64 (0.018*)

Min: Minimum, Max: Maximum, SD: Standard Deviation, IQR: Interquartile Range, χ^2^: chi-square test.

U: Mann-Whitney test, *p*: *p*-value, *: statistically significant.

### The correlation between knowledge and practice of the participants

**Fig. 4:**
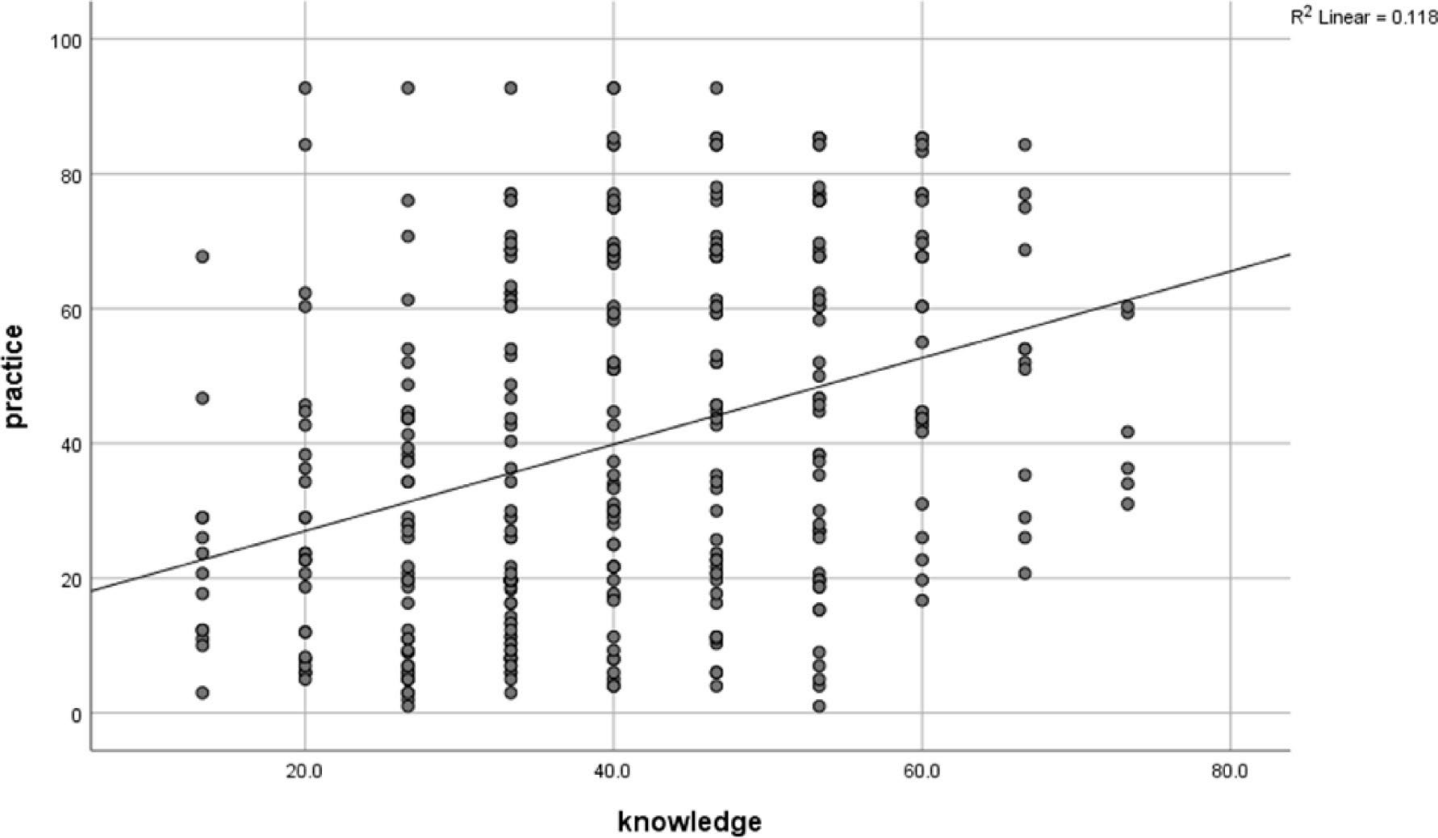
Correlation between knowledge and practice among participants regarding infection control precautions against blood-borne diseases, 2024 (*n* = 376).

After analyzing the data, a weak but significant positive correlation was found between knowledge of infection control precautions and actual practices (Spearman’s correlation coefficient (r) = 0.363, *p* < 0.001). This suggests that individuals with a higher level of knowledge about infection control precautions were more likely to practice them. (Fig. 4)

### The predictors of knowledge and practice regarding infection control precautions against blood-borne diseases

Table 3. presents the results of the generalized linear model regression analysis, which aimed to identify factors influencing participants’ knowledge and practices regarding infection control precautions against blood-borne diseases. The Omnibus test revealed good significant regression model for knowledge (χ² = 120.9, *p* < 0.001) and practices (χ² = 183.3, *p* < 0.001).

**Table 3:** The generalized linear regression model showing the predictors of knowledge and practice scores regarding infection control precautions against blood-borne diseases, 2024 (n= 376).

	**Knowledge**				**Practice**			
		**Wald Chi-Square**	**df**	**Sig.**	**B**	**Wald Chi-Square**	**df**	**Sig.**
(Constant)		91.231	1	0.000		53.662	1	0.000
Sex		42.117	47	0.675		60.477	47	0.090
Age		0.680	2	0.712		3.431	2	0.180
Living governorate		0.028	1	0.867		0.181	1	0.670
Nature of residence		0.770	1	0.380		0.203	1	0.653
Education level		22.702	4	0.001*		11.271	4	0.024*
·Primary school	4.424	3.664	1	0.056	9.447	5.630	1	0.018*
·Secondary school	6.322	8.766	1	0.003*	8.535	5.384	1	0.020*
·Bachelor degree	8.291	8.763	1	0.003*	9.151	3.598	1	0.058
·Post graduated	22.113	9.713	1	0.002*	11.819	0.935	1	0.334
·Read and write ^R^	-	-	-	-	-	-	-	-
Occupation		7.099	2	0.029*		71.936	2	0.001*
·Manual job	-4.040	6.340	1	0.012*	-21.814	62.291	1	0.001*
·Professional job	-0.703	6.340	1	0.764	-2.427	0.363	1	0.547
·Not working ^R^	-	-	-	-	-	-	-	-
Marital status		0.638	2	0.727		1.676	2	0.432
Sources of information		7.523	6	0.275		6.919	6	0.328
Duration of HCV recovery		0.960	2	0.619		3.759	2	0.153
Taking the HBV vaccine		10.952	2	0.004*		1.944	2	0.378
·I don’t know	-5.261	10.934	1	0.001*				
·No	-1.475	0.837	1	0.350				
·Yes^R^	-	-	-	-				

B: B coefficients, df: degree of freedom, sig.: significance, ^*^ : *p*-value significant at ˂ 0.05, ^R^: reference

Regarding knowledge, the regression model revealed that education level, occupation, and status of taking the HBV vaccine were significant predictors. Higher education levels were associated with increased knowledge scores. Compared to those with only basic reading and writing skills, individuals with secondary education had a 6.322-point increase in knowledge scores (*p* = 0.003), those with university degrees had an 8.291-point increase (*p* = 0.003), and those with postgraduate studies had a 22.113-point increase (*p* = 0.002). Individuals working in manual labor had significantly lower knowledge scores (4.040 points lower, *p* = 0.012) compared to unemployed individuals. HBV vaccination status also affected the knowledge status significantly (*p *= 0.004). Individuals unsure of their HBV vaccination status had significantly lower knowledge scores (−5.261 points lower, *p* = 0.001) compared to those who received the HBV vaccine.

Regarding practice scores, education level and occupation were also significant predictors. Compared to those with only basic reading and writing skills, individuals with primary education had a 9.447-point increase in practice scores (*p* = 0.018), and those with secondary education had an 8.535-point increase (*p* = 0.02). Individuals working in manual labor had significantly lower practice scores (21.814 points lower, *p* = 0.001) compared to unemployed individuals.

## Discussion

Following the success of Egypt’s nationwide HCV treatment campaign, the risk of reinfection has become a significant concern. This study aimed to assess the knowledge and practices regarding infection control precautions among individuals who have recovered from hepatitis C. The current study found that 73.9% of respondents demonstrated poor knowledge of infection control precautions against blood-borne diseases, which contrasts with findings from a study in British Columbia, Canada, where patients treated with DAAs exhibited good knowledge about HCV and its modes of transmission^22^. Our findings are in line with a study in New Zealand, which found limited knowledge of HCV among a middle-aged population^23^. It also further aligns with a study among Egyptian dental students, which reported low knowledge scores regarding infection control precautions against blood-borne infections^24^. Similarly, Sultan et al. (2018) found that more than half of HCV-infected Egyptians had low knowledge levels^25^. The persistent knowledge gaps observed across the Egyptian studies highlight an ongoing risk of reinfection despite successful treatment campaigns.

Only 36.4% of respondents in the present study demonstrated good infection control practices, lower than rates observed in other studies. A community-based study in Ethiopia reported good infection control practices among 50.3% of the general population regarding hepatitis B and C infections^26^. A systematic review and meta-analysis found satisfactory standard precautions among HCWs in Low- and Middle-Income Countries (LMICs) in 53% of cases^27^. Studies among dental students in Egypt^24^ and nurses and midwives in Sudan^28^ also reported higher rates of good infection control practices compared to our findings. These findings suggest a concerning level of suboptimal infection control practices among recovered HCV patients in the current study population.

The present study found that the highest percentage of good practice was for washing the injured area with soap and water after needle stick injuries (NSI), with 84.3% of respondents adhering to this practice. This finding aligns with a previous study in Sudan, which reported 82.5% of subjects washing their hands with soap and water after NSI^28^, and exceeds the findings of a study in China, where 69% of participants cited washing wounds with soap and water^29^. In contrast, the lowest adherence was observed for proper sharps disposal and safe blood spill cleanup. These findings are supported by a previous systematic review, which reported that the majority of diabetic patients lack proper sharps waste disposal practices^30^, and a cross-sectional Ghanaian study that reported that 72% of respondents did not promptly wipe up blood spills^31^.

The present study found that healthcare professionals have better knowledge and practice of infection control compared to non-healthcare participants. This aligns with existing literature, as healthcare workers generally possess a higher level of awareness due to formal training and consistent exposure to infection risks. However, even with this advantage, our findings reveal a significant gap: a suboptimal level of knowledge and practice among the healthcare group. This is consistent with previous studies in both middle- and low-income countries that have also identified deficiencies in infection control among healthcare workers, particularly in areas like hand hygiene, proper use of PPE, and adherence to standardized protocols^32–35^.

The current study found a weak but significant positive correlation between knowledge of infection control precautions and actual practices (*r* = 0.363, *p* < 0.001), consistent with findings from an Indian study that also demonstrated a positive correlation between knowledge and practices (*r* = 0.146, *p* < 0.05)^36^. Increased knowledge of nurses regarding patient safety significantly enhances their compliance with practices that promote the prevention of bloodborne pathogens^37^. This suggests that individuals with greater knowledge of infection control precautions are more likely to implement them in practice.

The regression model in the present study revealed that higher education levels and professional occupations were significant predictors of higher knowledge and practice scores. In the same way, a study in New Zealand identified higher levels of qualification and occupation sector as predictors of higher knowledge scores regarding HCV infection^23^. Similarly, an Egyptian study found that higher respondent knowledge and practices levels were associated with higher occupational status and education levels^38^. Higher education generally provides individuals with better access to information and critical thinking skills, enabling them to better understand, retain, and implement information about health-related topics, including infection control measures. Professional occupations prioritize health and safety training, which often includes instruction on infection control measures, significantly improving knowledge and practices among individuals in these professions^23,38^.

The status of taking the HBV vaccine also significantly influenced the knowledge level, according to our findings, as individuals unsure of their HBV vaccination status had significantly lower knowledge scores compared to those who confirmed receiving the HBV vaccine. This result may suggest that the act of receiving the HBV vaccine itself serves as an educational experience. The vaccination process often involves pre-vaccination counseling, which can increase awareness of blood-borne diseases and the importance of infection control measures. Furthermore, receiving the HBV vaccine frequently involves visits to healthcare settings, exposing individuals to infection control practices in action, such as the use of personal protective equipment by healthcare workers^39,40^.

While HBV vaccination is crucial for HCV-recovered patients, only 47.9% of participants in this study had received the vaccine. This rate is lower than that observed among dental students in Yemen (71.7%)^41^ but higher than the vaccination coverage among rural adults in China (20.2%)^42^. Dual HBV and HCV infection is a significant public health concern, as both viruses share common transmission routes. Notably, approximately 25% of HCV-positive individuals exhibit markers of past or present HBV infection. Furthermore, HBV reactivation can occur during HCV treatment with DAAs. Besides, many HCV-treated patients continue to engage in high-risk behaviors, increasing their susceptibility to reinfection. Given the high prevalence of co-infection and the risk of HBV reactivation, HBV vaccination is crucial for preventing HBV infection in HCV-infected or treated individuals^43^.

Egypt has successfully reduced its HCV prevalence from 7% in 2014 to just 0.38% in 2023, giving an example of the effectiveness of a broad, nationwide treatment campaign that aligns with the WHO 2030 elimination agenda^3,4^. Another successful program was that in Iceland, which had the “Treatment as Prevention” (TasP) initiative. Iceland’s strategy focused on a combination of universal screening, simplified protocols, and accessible care for all, including high-risk groups. This allowed them to treat over 80% of their infected population within two years, with a 92.4% cure rate, showing that elimination is an achievable goal. Both broad and targeted programs highlight four essential components for success: elevated public awareness, effective screening, affordable access to DAAs, and ongoing public health support. By integrating these key elements into its ongoing strategy, any country with a high prevalence of HCV can guarantee effective elimination of HCV^44,45^.

Although Egypt has achieved remarkable success in treating millions with HCV, our study reveals a significant public health challenge: a substantial portion of recovered patients lack adequate knowledge and practice of basic infection control measures, even among healthcare professionals. This persistent gap, especially among individuals with lower education and manual labor jobs, poses a major obstacle to the sustained elimination of the virus and challenges the country’s ability to meet the WHO 2030 viral hepatitis elimination strategy. Our findings provide actionable, evidence-based insights by pinpointing specific socio-demographic factors like education, occupation, and HBV vaccination status that are critical for designing effective, targeted interventions. The discovery of persistent knowledge gaps and suboptimal practices in a post-treatment setting serves as an urgent signal, demonstrating that the campaign success is not self-sustaining. Targeted health education campaigns should address common misconceptions about HCV transmission, such as the belief that it spreads through handshakes or simple contact. These campaigns should prioritize the importance of hand hygiene, safe needle handling, and other sound infection control precautions. By addressing these critical areas, public health efforts can effectively support the long-term health and well-being of recovered HCV patients and contribute to the ongoing fight against blood-borne diseases, especially in countries with high prevalence of these infections, such as Egypt.

### Limitations and points of strength

This study has several limitations. First, the cross-sectional design precludes the establishment of cause-and-effect relationships between variables. Second, the use of non-probability sampling methods (convenience and snowball sampling) may have introduced selection bias, potentially limiting the generalizability of the findings to the broader population. Third, the reliance on self-reported data without an observational checklist may be subject to recall bias and social desirability bias, where participants might report what they think is the “correct” answer rather than their actual practices. Finally, the study focused on a specific population in Egypt, which may limit the applicability of the findings to other regions or populations with different socio-cultural contexts. This study strengths lie in its focus on the crucial public health issue of preventing HCV reinfection following Egypt highly successful national campaign to detect and treat HCV-infected individuals, a significant achievement considering the country previously high prevalence of the virus. To our knowledge, this is one of the first studies to specifically assess the knowledge and practices of HCV-recovered patients regarding precautions against blood-borne diseases in Egypt following the nationwide mass treatment campaign. This provides critical, real-world data on a previously unexamined population, directly addressing a key public health question about the risk of reinfection. A structured interview questionnaire and asking the questions in the same manner were used to minimize interviewer bias and facilitate systematic data collection on socio-demographic characteristics, knowledge, and practices. Rigorous methodological steps, including a pilot study, assessments of face and content validity, and reliability testing, enhanced the questionnaire quality. Furthermore, the study employed a comprehensive analytical approach, utilizing descriptive statistics, correlation analysis, and regression analysis to thoroughly examine the data and identify key associations.

## Conclusions

The present study found significant knowledge gaps and suboptimal practices regarding infection control among participants, particularly concerning specific modes of HCV transmission. While healthcare professionals showed better knowledge and practice than others, their levels were still suboptimal. The study reported that education level and occupation were significant predictors of knowledge and practice. Status of HBV vaccination was a significant predictor of knowledge. Subsequently, the study recommends enhancing knowledge and promoting safe practices to reduce the risk of HCV reinfection and to combat blood-borne diseases in Egypt.

## Supplementary Information

Below is the link to the electronic supplementary material.

Supplementary Material 1

Supplementary Material 2

## Data Availability

The datasets used and/or analyzed during the current study are available from the corresponding author on reasonable request.

## Abbreviations

CDC: The Centers for Disease Control and Prevention
DAAs: Direct-Acting Antiviral medications
HBV: Hepatitis B Virus
HCV: Hepatitis C Virus
IQR: Interquartile Range
IPC: Infection Prevention and Control
LMICs: Low and Middle-Income Countries
MOHP: The Egyptian Ministry of Health and Population
NSI: Needle Stick Injuries
*p*: *p*-value
PPE: Personal Protective Equipment
R: The Spearman Correlation Coefficient
SD: Standard Deviation
TasP: Treatment as Prevention
U: Mann-Whitney test
χ2: Chi-square test
