# Supplementary material for: Knowledge and practices regarding infection control precautions against blood-borne diseases among recovered HCV patients in Egypt
Source: Sci Rep. 2025 Oct 31;15:38108. doi: 10.1038/s41598-025-23618-3 (PMC12578818; doi:10.1038/s41598-025-23618-3)
Supplement: Supplementary file 1 — Supplementary Material 1 [file 41598_2025_23618_MOESM1_ESM.pdf]

# Knowledge and practices of recovered HCV patients regarding infection control precautions against blood-borne diseases in Egypt: A cross-sectional study

**Journal name:** Scientific Reports

**Authors:** Mohamed Fakhry Hussein*, Wesal Youssef Hassan, Mohamed Hossam Mohamed, Marwa Mostafa Mohamed, Hossam Mohamed Hassan Soliman

**Corresponding author:** Mohamed Fakhry Hussein: Department of Occupational Health and Industrial Medicine, High Institute of Public Health, Alexandria University, Alexandria, Egypt. Email: hiph-mohamedfakhry@alexu.edu.eg

You are invited to participate in research entitled "Knowledge and practices of recovered HCV patients regarding infection control precautions in Egypt". This information is crucial in understanding the level of awareness about infection control concepts and the infection control practices in the different activities related to exposure to blood in HCV-recovered persons.

Your participation is voluntary and anonymous. You are free to withdraw at any time. This study was approved by the Ethics Committee of the Ministry of Health and Population. The information provided will be used only for research purposes. Confidentiality will be maintained. The survey should take about 5 to 10 minutes to complete. Thank you for sharing your valuable time with us.

## Code number of the subject:

## Please answer the following socio-demographic questions

1. **Gender or sex:**
   1. Male
   2. Female

2. **Age in years:**

3. **Now I am living in:**

   1) Alexandria
   2) El Behera
   3) Cairo
   4) Giza
   5) Marsa Matrouh
   6) Kafr El Sheikh
   7) Others

4. **Place of residence:**
   1. Urban area
   2. Rural area

5. **Level of education completed:**
    1. I did not complete any level of education.
    2. Primary education
    3. Secondary education
    4. University education
    5. Post graduated.

6. **Social status:**
    1. Married
    2. Single
    3. Widow
    4. Divorced

7. **Occupation:**
    1. Medical field or paramedical eld like physician, nurse, midwife, or healthcare
    2. Engineer
    3. Chemist
    4. Manager
    5. Clerk
    6. Farmer
    7. Miner
    8. Herdsman
    9. Fisherman
    10. Service and sales workers
    11. Skilled technical worker
    12. Elementary occupations
    13. Trader
    14. Student
    15. Not working/retired
    16. Others

8. **Source of Information about infection control precautions against blood-borne diseases:**
    1. Health worker
    2. Mass media
    3. Community leaders
    4. Friends or neighbors
    5. Family member
    6. Scientific books or scientific websites
    7. I did not hear about it before

9. **Duration of HCV recovery:**

1. Since less than 1 year
2. For 1 year
3. For 2 years
4. For 3 years
5. For 4 years
6. For 5 years
7. More than 6 years

**10. Did you take the HBV vaccine?**

1. Yes
2. No
3. I do not know

**11. How many times have you been exposed to injury by sharp instruments within the last year?**

**Questions about your knowledge of infection control precautions against blood-borne diseases.**

**12. Blood-borne pathogens are microorganisms present in human blood that can cause disease.**

1. Yes
2. No
3. I do not know

**13. HBV, HCV, and HIV are examples of blood-borne infectious diseases.**

1. Yes
2. No
3. I do not know

**14. Blood transfusion could transmit blood-borne infections.**

1. Yes
2. No
3. I do not know

**15. Unsterilized instruments in Dental clinics could transmit blood-borne infection.**

1. Yes
2. No
3. I do not know

**16. Sharing shaving instruments, razors, or toothbrushes could transmit blood-borne infections.**
1. Yes
2. No
3. I do not know

**17. Needle stick injuries could transmit blood-borne infections.**
1. Yes
2. No
3. I do not know

**18. Exposure to patient blood or other body fluid could transmit blood-borne infections.**
1. Yes
2. No
3. I do not know

**19. Shaking hands with someone who has HCV could transmit the infection.**
1. Yes
2. No
3. I do not know

**20. HCV can be transmitted by working with someone who has HCV.**
1. Yes
2. No
3. I do not know

**21. There is a vaccination for HCV.**
1. Yes
2. No
3. I do not know

**22. Washing hands with soap and water thoroughly could prevent the transmission of blood-borne pathogens.**
1. Yes
2. No
3. I do not know

**23. Wearing gloves before contacting any blood spills could prevent the transmission of blood-borne pathogens.**
1. Yes
2. No
3. I do not know

24. **Wearing gloves before dealing with injection could prevent the transmission of blood-borne pathogens.**
    1. Yes
    2. No
    3. I do not know

25. **Avoiding needle stick and sharps injuries could prevent the transmission of blood-borne pathogens.**
    1. Yes
    2. No
    3. I do not know

26. **Testing blood and other body fluids for blood-borne pathogens is a must before dealing with them.**
    1. Yes
    2. No
    3. I do not know

**Questions about your behavior about dealing with sharps or blood.**

27. **If I am exposed to a needle stick injury or an injury with a sharp object, I will wash the injured part with soap and water.**
    1. Always
    2. Often
    3. Sometimes
    4. Rarely
    5. Never

28. **If I am exposed to blood splashing on the nose, mouth, eye, or skin, I will flush these parts with water.**
    1. Always
    2. Often
    3. Sometimes
    4. Rarely
    5. Never

29. **If I am exposed to a needle stick injury or injury with a sharp object, I will seek medical treatment immediately.**
    1. Always
    2. Often
    3. Sometimes
    4. Rarely
    5. Never

**30. I use sharps containers that are labeled and puncture-resistant to discard syringes or sharp instruments.**
1. Always
2. Often
3. Sometimes
4. Rarely
5. Never

**31. I Place sharps in sharps containers immediately after use.**
1. Always
2. Often
3. Sometimes
4. Rarely
5. Never

**32. I bend, recap, or break needles before discarding them.**
1. Always
2. Often
3. Sometimes
4. Rarely
5. Never

**33. I use my own instrument in the barbershop while cutting my hair.**
1. Always
2. Often
3. Sometimes
4. Rarely
5. Never

**34. I use my own razor.**
1. Always
2. Often
3. Sometimes
4. Rarely
5. Never

**35. I use my own shaving instruments (for men).**
1. Always
2. Often
3. Sometimes
4. Rarely
5. Never

   (-1) Female

**36. When there is a risk of exposure to blood, like giving injections or handling needles, I wear gloves.**
1. Always

2. Often
3. Sometimes
4. Rarely
5. Never

**37. I ask about instrument sterilization in the dental care clinic before any dental procedure.**

1. Always
2. Often
3. Sometimes
4. Rarely
5. Never

**38. I clean blood spills with my bare hands.**

1. Always
2. Often
3. Sometimes
4. Rarely
5. Never

**Thanks for your time**
