# Supplementary material for: Knowledge and practices regarding infection control precautions against blood-borne diseases among recovered HCV patients in Egypt
Source: Sci Rep. 2025 Oct 31;15:38108. doi: 10.1038/s41598-025-23618-3 (PMC12578818; doi:10.1038/s41598-025-23618-3)
Supplement: Supplementary file 2 — Supplementary Material 2 [file 41598_2025_23618_MOESM2_ESM.pdf]

Knowledge and practices of recovered HCV patients regarding infection control precautions against blood-borne diseases in Egypt: A cross-sectional study

Journal name: Scientific Reports

Authors: Mohamed Fakhry Hussein*, Wesal Youssef Hassan, Mohamed Hossam Mohamed, Marwa Mostafa Mohamed, Hossam Mohamed Hassan Soliman

Corresponding author: Mohamed Fakhry Hussein: Department of Occupational Health and Industrial Medicine, High Institute of Public Health, Alexandria University, Alexandria, Egypt. Email: hiph-mohamedfakhry@alexu.edu.eg

Table 1: Questions about the knowledge of infection control precautions against blood-borne diseases, 2024 (n=376)

Questions		No.	(%)	Mean	SD	Rank
Blood borne pathogens are microorganisms present in human blood that can cause disease	• Yes	204	54.3	1.67	0.808	12
	• No	91	24.2			
	• I don't know	81	21.5			
HBV, HCV, and HIV are examples of blood-borne infectious diseases.	• Yes	225	59.8	1.62	0.818	13
	• No	70	18.6			
	• I don't know	81	21.5			
Blood transfusion could transmit blood-borne infections.	• Yes	272	72.3	1.41	0.714	14
	• No	54	14.4			
	• I don't know	50	13.3			
Unsterilized instruments in Dental clinics could transmit blood-borne infection.	• Yes	173	46.0	1.91	0.911	4
	• No	62	16.5			
	• I don't know	141	37.5			
Sharing shaving instruments, razors, or toothbrushes could transmit blood-borne infections.	• Yes	177	47.1	1.91	0.919	4
	• No	56	14.9			
	• I don't know	143	38.0			
Needle stick injuries could transmit blood-borne infections.	• Yes	177	47.1	1.83	0.861	7
	• No	87	23.1			
	• I don't know	112	29.8			
Exposure to patient blood or other body fluid could transmit	• Yes	180	47.9	1.74	0.794	10
	• No	114	30.3			

blood-borne infections.	• I don't know	82	21.8			
Shaking hands with someone who has HCV could transmit the infection.	• Yes	124	33.0	1.94	0.772	3
	• No	151	40.1			
	• I don't know	101	26.9			
HCV can be transmitted by working with someone who has HCV.	• Yes	151	40.2	1.86	0.807	5
	• No	125	33.2			
	• I don't know	100	26.6			
There is a vaccination for HCV.	• Yes	166	44.1	1.84	0.837	6
	• No	104	27.7			
	• I don't know	106	28.2			
Washing hands with soap and water thoroughly could prevent the transmission of blood borne pathogens	• Yes	151	40.2	1.98	0.887	2
	• No	81	21.5			
	• I don't know	144	38.3			
Wearing gloves before contacting any blood spills could prevent the transmission of blood borne pathogens	• Yes	177	47.1	1.83	0.865	7
	• No	85	22.6			
	• I don't know	114	30.3			
Wearing gloves before dealing with injection could prevent the transmission of blood borne pathogens.	• Yes	170	45.2	1.71	0.732	11
	• No	144	38.3			
	• I don't know	62	16.5			
Avoiding needle stick and sharps injuries could prevent the transmission of blood borne pathogens	• Yes	142	37.8	2.03	0.889	1
	• No	79	21.0			
	• I don't know	155	41.2			
Testing blood and other body fluids for blood borne pathogens is a must before dealing with them	• Yes	191	50.8	1.78	0.865	9
	• No	77	20.5			
	• I don't know	108	28.7			

Table 2: Questions about the practice of infection control precautions against blood-borne diseases, 2024 (n=376)

Questions		No.	(%)	Mean	SD	Rank
If I am exposed to a needle stick injury or an injury with a sharp object, I will wash the injured part with soap and water	• Always	317	84.3	1.37	0.926	11
	• Often	11	2.9			
	• Sometimes	30	8.0			
	• Rarely	9	2.4			
	• Never	9	2.4			
If I am exposed to blood splashing on the nose, mouth, eye, or skin, I will flush these parts with water.	• Always	95	25.3	2.39	1.694	7
	• Often	188	50.0			
	• Sometimes	11	2.9			
	• Rarely	62	16.5			
	• Never	20	5.3			
If I am exposed to a needle stick injury or injury with a sharp object, I will seek medical treatment immediately.	• Always	54	14.4	2.35	1.498	8
	• Often	172	45.7			
	• Sometimes	55	14.6			
	• Rarely	51	13.6			
	• Never	44	11.7			
I use sharps containers that are labeled and puncture-resistant to discard syringes or sharp instruments	• Always	55	14.6	2.31	1.603	9
	• Often	205	54.5			
	• Sometimes	11	2.9			
	• Rarely	33	8.8			
	• Never	72	19.1			
I Place sharps in sharps containers immediately after use.	• Always	27	7.2	2.12	1.337	10
	• Often	179	47.6			
	• Sometimes	34	9.0			
	• Rarely	81	21.5			
	• Never	55	14.6			
I bend, recap, or break needles before discarding them	• Always	61	16.2	2.43	1.549	6
	• Often	163	43.4			
	• Sometimes	25	6.6			
	• Rarely	69	18.4			
	• Never	58	15.4			
I use my own instrument in the barbershop while cutting my hair.	• Always	112	29.8	2.85	1.676	3
	• Often	122	32.4			
	• Sometimes	16	4.3			
	• Rarely	81	21.5			
	• Never	45	12.0			

I use my own razor	• Always	73	19.4	2.63	1.583	4
	• Often	147	39.1			
	• Sometimes	43	11.4			
	• Rarely	53	14.1			
	• Never	60	16.0			
I use my own shaving instruments (for men).	• Always	50	13.3	2.47	1.533	5
	• Often	161	42.8			
	• Sometimes	22	5.9			
	• Rarely	60	16.0			
	• Never	83	22.1			
When there is a risk of exposure to blood, like giving injections or handling needles, I wear gloves.	• Always	118	31.4	3.11	1.63 5	2
	• Often	106	28.2			
	• Sometimes	41	10.9			
	• Rarely	47	12.5			
	• Never	64	17.0			
I ask about instrument sterilization in the dental care clinic before any dental procedure	• Always	63	16.8	2.63	1.56 5	4
	• Often	145	38.6			
	• Sometimes	29	7.7			
	• Rarely	58	15.4			
	• Never	81	21.5			
I clean blood spills with my bare hands.	• Always	208	55.3	3.77	1.558	1
	• Often	54	14.4			
	• Sometimes	27	7.2			
	• Rarely	52	13.8			
	• Never	35	9.3			
